# Simultaneous Measurement of Perfusion and T_2_* in Calf Muscle at 7T with Submaximal Exercise using Radial Acquisition

**DOI:** 10.1038/s41598-020-63009-4

**Published:** 2020-04-14

**Authors:** Sultan Z. Mahmud, L. Bruce Gladden, Andreas N. Kavazis, Robert W. Motl, Thomas S. Denney, Adil Bashir

**Affiliations:** 1 Department of Electrical and Computer Engineering, Auburn University, Auburn, AL 36849 USA; 2 School of Kinesiology, Auburn University, Auburn, AL 36849 USA; 3 Department of Physical Therapy, University of Alabama at Birmingham, Birmingham, AL 35294 USA

**Keywords:** Imaging, Biomedical engineering, Electrical and electronic engineering, Biological techniques, Biotechnology, Medical research

## Abstract

Impairments in oxygen delivery and consumption can lead to reduced muscle endurance and physical disability. Perfusion, a measure of microvascular blood flow, provides information on nutrient delivery. T_2_* provides information about relative tissue oxygenation. Changes in these parameters following stress, such as exercise, can yield important information about imbalance between delivery and consumption. In this study, we implemented novel golden angle radial MRI acquisition technique to simultaneously quantify muscle perfusion and T_2_* at 7T with improved temporal resolution, and demonstrated assessment of spatial and temporal changes in these parameters within calf muscles during recovery from plantar flexion exercise. Nine healthy subjects participated the studies. At rest, perfusion and T_2_* in gastrocnemius muscle group within calf muscle were 5 ± 2 mL/100 g/min and 21.1 ± 3 ms respectively. Then the subjects performed plantar flexion exercise producing a torque of ~8ft-lb. Immediately after the exercise, perfusion was elevated to 79.3 ± 9 mL/100 g/min and T_2_* was decreased by 6 ± 3%. The time constants for 50% perfusion and T_2_* recovery were 54.1 ± 10 s and 68.5 ± 7 s respectively. These results demonstrate successful simultaneous quantification of perfusion and T_2_* in skeletal muscle using the developed technique.

## Introduction

Muscle weakness and fatigue are common manifestations of a variety of diseases that decrease mobility such as multiple sclerosis (MS), Parkinson’s disease (PD), and aging^1–4^. The underlying causes of muscle weakness and fatigue are not completely understood. It has been shown that impaired cellular energy production plays an important role in muscle fatigue^5^. Cells require adequate supplies of oxygen (O_2_) and nutrients for energy production, and deficiency in O_2_ supply and extraction may reduce cellular energy production required for muscle function. Accordingly, measurements of O_2_ delivery and microvascular oxygenation are of considerable interest and have the potential to provide useful understanding of the biochemical mechanisms of decreased mobility in disease and the assessment of treatment targets and responses.

Perfusion is the delivery of tissue mass normalized rate of blood, which also accommodates the supply of O_2_ and other nutrients to the tissue. Quantitative perfusion in skeletal muscle can be measured non-invasively using the arterial spin labeling (ASL) MRI technique. This technique has been used to study muscle perfusion in humans with ischemia- in reperfusion and exercise paradigms^6,7^. These studies have demonstrated that skeletal muscle perfusion dynamics are capable of providing insights into pathological and physiological functions^8–10^.

Blood oxygenation level-dependent (BOLD) contrast has been used to infer changes in blood oxygenation in skeletal muscle in response to various stimuli^11^. Muscle BOLD contrast results primarily from changes in the total amount of deoxyhemoglobin in the imaging volume^12^. The decrease in the deoxyhemoglobin content in a voxel causes a decrease in susceptibility hence increased signal intensity in the gradient echo BOLD MRI images. The BOLD signal largely depends on the changes in T_2_*. Quantitative T_2_* maps may provide more specific physiological information about muscle oxygen extraction in response to stimuli.

Oxygen delivery and demand are tightly coupled in exercising muscle and this coupling might be compromised in pathologic state^13^. Combined measurement of dynamic perfusion and T_2_* in skeletal muscle has the potential to provide important information about oxidative metabolism and functional deficits. Studies of combined quantification of perfusion and T_2_* or other markers of tissue oxygenation have been implemented for reactive hyperemia^6,14,15^, but there has been no demonstration of this technique for submaximal exercise (functional hyperemia) at 7T using radial acquisition. We chose functional hyperemia because it is more physiologically analogous to the demands of daily living than reactive hyperemia. High signal to noise advantage of 7T and low susceptibility to bulk motion due to radial acquisition will allow more robust determination of perfusion and T_2_* in dynamic studies with higher temporal resolution. Although several challenges, such as magnetic field inhomogeneity, need to be overcome at ultra-high filed studies, but improved SNR and improved contrast at ultra-high field benefits the dynamic measurement of ASL and T_2_*.

The main goal of this study is to develop and demonstrate the feasibility of a technique to simultaneously determine dynamic changes in perfusion and T_2_* in calf muscle at improved temporal resolution with an exercise stimulus. High temporal resolution is important for detection of significant changes in these metabolism related parameters following exercise. To accomplish this aim, we developed a novel interleaved golden angle radial MRI pulse sequence and quantified muscle perfusion and T_2_*. We built an MRI compatible plantar flexion exercise device and demonstrated the assessment of spatial and temporal changes in these parameters in calf muscle during recovery from plantar flexion exercise. Combined measurements can not only reduce experiment time but also several types of information can be obtained with same stimulus.

## Methods

The study was approved by the Auburn University Institutional Review Board (IRB). All experiments were performed in accordance with the IRB guidelines and regulations. Informed consent was provided by each subject prior to participation in the study.

### Recruitment

All experiments were done on a Siemens 7T Magnetom (Erlangen, Germany) using a surface coil. Nine healthy volunteers (six females), age = 28 ± 12 years, and weight = 67 ± 8 kg, participated in the study. Inclusion criteria was normal range of motion (> 20°) for ankle joint flexion.

### Imaging sequence

Flow alternating inversion recovery (FAIR) is an established ASL method in which slice-selective (SS) and non-selective (NS) inversion pulses are used to acquire tag and control image^16^. Saturation inversion recovery (SATIR) is a variant of FAIR with short recovery time acquisition, thus allowing for fast dynamic measurements^17^. The dead time between tagging and acquisition (post labeling delay (PLD)) during perfusion imaging can be used to interleave a gradient echo (GRE) imaging loop to acquire multi-echo images from a slice posterior to the perfusion imaging slice. These multi-echo GRE images are used to quantify T_2_*. This interleaved approach allows simultaneous measurement of perfusion and T_2_* with a single scan.

**Figure 1:**
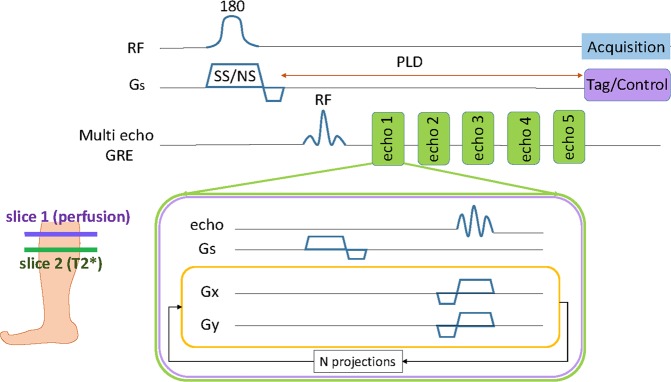
Schematic of pulse sequence and prescription of imaging slices. Pulse sequence consisted of two loops. The outer perfusion loop (top) was acquired with interleaved tagging (SS) and control (NS) adiabatic inversion pulse followed by PLD and radial acquisition of perfusion imaging slice (slice 1). Inner loop consisted of multi-echo GRE pulse sequence implemented during the PLD delay to get multi echo images for T_2_* calculation. Five echo images were acquired during one outer perfusion loop with echo times = 2.2, 5.0, 7.8, 10.6 and 13.4 ms. Perfusion slice was selected 3 cm proximal to the T_2_* slice which accounts for the incoming arterial blood flow direction.

Schematic of pulse sequence is shown in Fig. 1. ASL sequence SATIR was implemented using adiabatic full passage hyperbolic secant inversion pulse for spin tagging with golden angle radial readout (Fig. 1 - slice 1). The PLD was used for interleaved acquisition of T_2_* data for a slice located 3 cm distally from the perfusion slice (Fig. 1 - slice 2). A multi-echo GRE sequence with radial acquisition was used for T_2_* mapping. Temporal resolution for perfusion and T_2_* was approximately 1.3 seconds.

### Phantom experiment

Initial experiments were done in phantoms to optimize radial image reconstruction at 7T and validate measurements from the new sequence.

#### Shift estimation and correction

In radial acquisition, the read-out orientation is changed to different angles for each k-space line to acquire a set of radial projections. Gradient imperfection and eddy current effects cause delays in gradient timing and have an angle dependent effect on the projections. This causes the center of acquired projections to shift yielding artifacts in reconstructed images^18,19^.

We characterized these gradient imperfections in phantoms by collecting projections for x-gradient along angles 0° and 180° and y-gradient along angles 90° and 270°. A delay in gradient timing causes a spatial shift in two projections acquired with opposite gradient polarity. This shift was corrected as described below for x-axis.

Flip one of the 0° and 180° spokes.

1. Take the FT of the magnitude of two spokes.
2. Calculate and plot the phase difference between these two signals. The center of the plot will show a linear phase change due to spatial shift in projections.
3. Fit a linear equation to the phase difference and calculate the slope of that line.
4. Shift along k_y_ = 0 line was calculated from the slope from the equation given by:

$\Delta {k}_{x}=-\frac{slope\times N}{4\pi }$, where N = no. of samples.

Same procedure is used with 90° and 270° spokes to calculate Δk_y_ along k_x_=0 line. Shift along any arbitrary projection angle ϴ is calculated using^20^:

1 $$\Delta k(\theta )=\Delta {k}_{x}co{s}^{2}(\theta )+\Delta {k}_{y}co{s}^{2}(\theta )$$

Agarose phantoms at 0.5, 1, 2, 3, 4 and 5% concentration were prepared in 50 mL vials to validate perfusion and T_2_* measurements. The phantoms were prepared by mixing Agarose powder with water, then heated until boiling. New interleaved pulse sequence was used to determine T_2_* and perfusion and the results were compared to the results from vendor provided multi-echo GRE pulse sequence.

### In vivo study design

After scanner adjustments, perfusion and T_2_* data were acquired in the calves of the participants at rest. A ^1^H/^31^P surface coil (RAPID Biomedical, Rimpar, Germany) with single channel compatible with Siemens Magnetom 7T system was used for the study. The maximum average transmit power of ^1^H coil was 11.5 W with quadrature RF polarization as per specification. The mean coil dimension of ^1^H double-loop was 140 mm × 225 mm.

A 6 ms long slice selective (SS) and non-selective (NS) hyperbolic secant adiabatic inversion pulse was used to acquire tag and control images in an interleaved fashion. The inversion efficiency was tested in phantoms as well as *in vivo*, and a voltage of 500 V was selected for satisfactory inversion slice profile (Figs. S.1 and S.2 in Supplementary Information). Specific common acquisition parameters for perfusion and T_2_* were: FOV = 192 mm, slice thickness = 5 mm, TR = 1.28 s, flip angle = 15°, bandwidth = 500 Hz/pixel and 64 radial projections. Perfusion PLD = 1 s. TE = 2.2 ms for perfusion and TE = 2.2, 5.0, 7.8, 10.6 and 13.4 ms for GRE sequence.

The subjects then performed 2 minutes of plantar flexion at 0.5 Hz using a home-built MRI compatible exercise device. The knee angle was 0° (aligned with the scanner table) during exercise and the leg was held in place with Velcro straps to prevent bulk motion. The paddle of the device was strapped to an adjustable support with a rubber band and the subjects pushed against the generated resistance. The support position can be adjusted to produce different torques. The torque produced by the subjects in this study was measured to be ~8 ft-lb (~10.8 Nm). A metronome was used to provide audio signal to the volunteers for guiding the ankle plantar flexion exercise protocol. Only slice-selective images were acquired for 3 minutes as participants rested and recovered from exercise, since non-selective acquisitions will interfere with the nearby T_2_* slice.

It can be shown from SATIR signal derivation^17^ that

2 $${M}_{SS}(t)+{M}_{NS}(t)={M}_{0}(2-2{e}^{-\frac{t}{{T}_{1}}})$$

where, M_SS_ and M_NS_ are slice selective and non-selective acquisitions respectively, M_0_ is the tissue magnetization at equilibrium, T_1_ is tissue spin-lattice relaxation time constant. As SATIR approach assumes constant T_1_ for all perfusion values, Eq. (2) shows that, if the signals are corrected for the T_2_* weighting, the summation of SS and NS signal is approximately constant regardless of the perfusion value. NS value during recovery period can be estimated by subtracting the recovery SS value from the (SS+NS) value at rest. Not acquiring NS images during recovery from exercise results in improved temporal resolution. The recovery NS signal can be estimated using

3 $${M}_{NS,rec}(t)=({M}_{SS,rest}+{M}_{NS,rest}){e}^{\left(\frac{1}{{T}_{2,rest}^{\ast }}-\frac{1}{{T}_{2,rec}^{\ast }}\right)t}-{M}_{SS,rec}(t)$$

where M_NS,rest_ and M_SS,rest_ are NS and SS acquisitions respectively at rest, M_NS,rec_ and M_SS,rec_ are NS and SS signals respectively during recovery post-exercise, T_2,rest_* and T_2,rec_* are T_2_* values at rest and during recovery respectively.

Perfusion weighted images were generated from the differences between slice selective and non-selective images at rest. The muscle area activated with high perfusion due to exercise was identified from the perfusion weighted images immediately after exercise. From this muscle activation map a region of interest (ROI) was selected to calculate the perfusion using^17^

4 $$f=-\frac{\lambda }{T}.\,\mathrm{ln}\left[\frac{{M}_{SS}(T)-{M}_{NS}(T)}{{M}_{SS}(T)+{M}_{NS}(T)}.(1-{e}^{\frac{T}{{T}_{1}}})+1\right]$$

where f is the perfusion (mL/100 g/min), λ is tissue partition coefficient (0.9 mL/g), T is tagging time (or PLD), T_1_ for tissue is 1.55 sec.^21^ at 7T. Dynamic change in perfusion was measured as a change from the resting perfusion during the recovery period.

T_2_* was calculated by fitting a mono-exponential function to the magnitude signal intensity from multi-echo data using

5 $$A={A}_{0}{e}^{-TE/{{T}_{2}}^{\ast }}$$

T_2_* was normalized to the resting T_2_* values for each individual subject. This was done to account for differences in T_2_* between subjects due to physiological variances or different shimming.

## Results

### Phantom experiment

**Figure 2:**
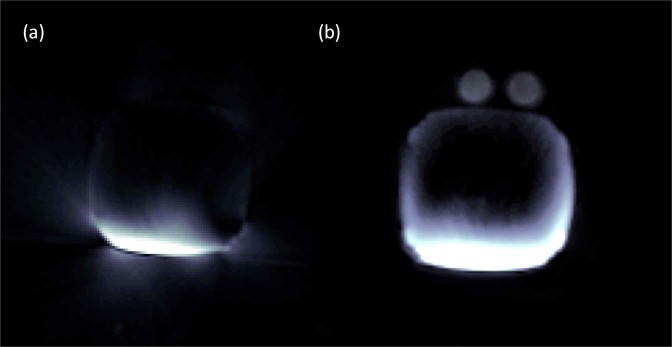
Difference between radial image reconstruction without and with gradient delay error correction. (**a**) Phantom image shows artifacts without the correction of gradient delay errors. There are two vials on the top that disappear in the reconstructed image. (**b**) Image after correction for gradient delays.

Radial image reconstruction without correction of gradient delay errors showed artifacts (Fig. 2a). Two vials placed on the upper side of a box-shaped phantom were completely missing in the reconstructed image without delay correction. 0° and 180° calibration projections resulted in a sample shift of 0.595 along k_x_ and 90° and 270^o^ projections resulted in a sample shift of 0.696 along k_y_. *In vivo* calibration data (Δk_x_ = 0.588 and Δk_y_ = 0.689) was similar to that from phantom experiments. The artifacts in the reconstructed image were removed and the vials were visible after accounting for gradient delay errors (Fig. 2b). This same protocol was used for all imaging studies *in vivo*.

### Phantom study

**Figure 3:**
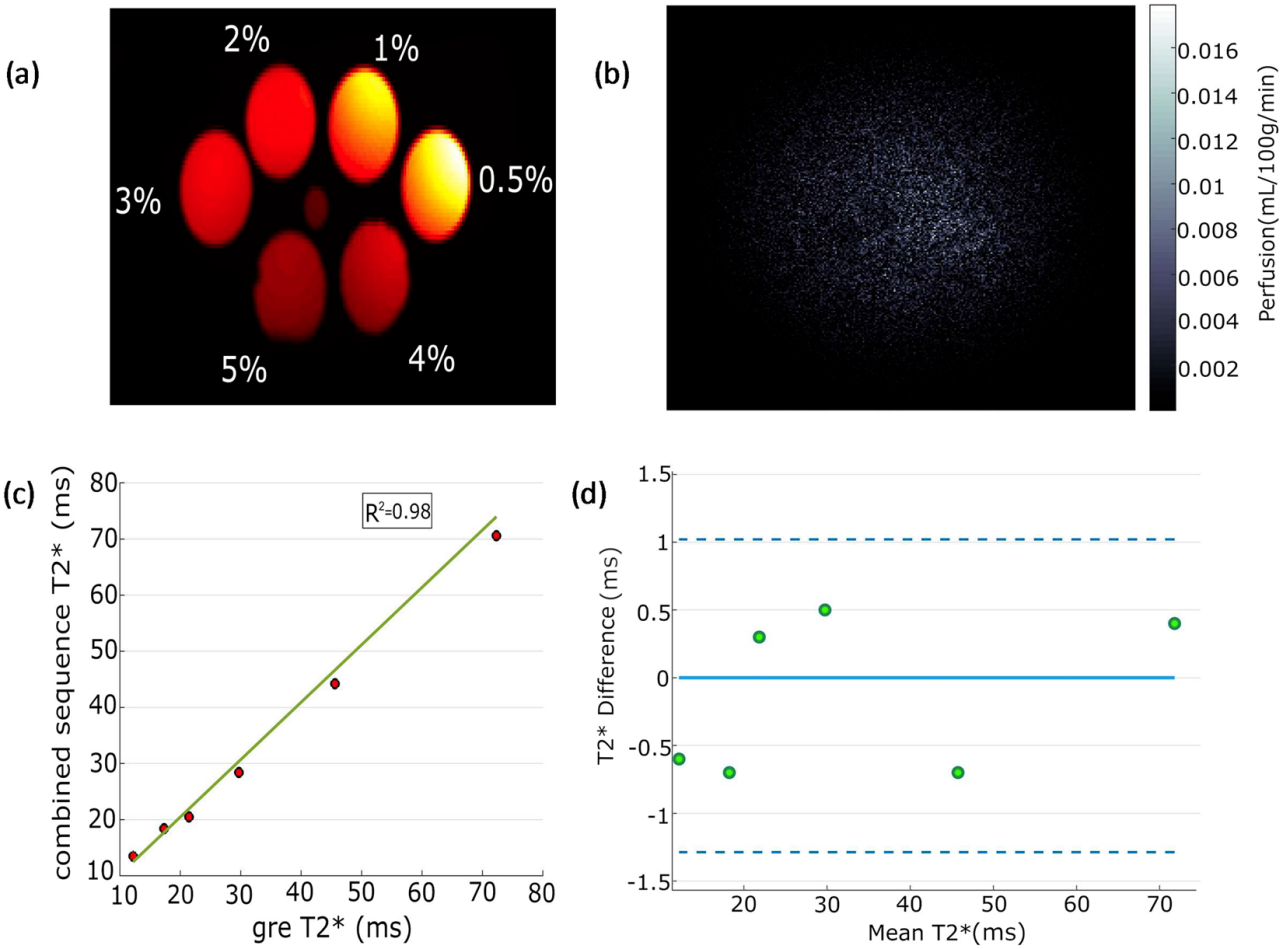
(**a**) Image of the phantom. (**b**) Perfusion map showing absence of perfusion in phantom. (**c**) Correlation plot showing excellent correlation between T_2_* calculated from new sequence and GRE sequence. (**d**) Bland-Altman plot also showing very good agreement between two sequences.

Figure 3 shows six agar phantoms with different concentrations. Perfusion image of the phantom shows noise indicating that the presence of second slice for T_2_* has no spillover effect on the perfusion slice. Excellent correlation was found between the two T_2_* values measured by standard multi-echo GRE sequence and new interleaved sequence with r^2^ = 0.98. The Bland-Altman plot also shows very good agreement between two methods with mean difference = −0.12 ± 1.15.

### In vivo

**Figure 4:**
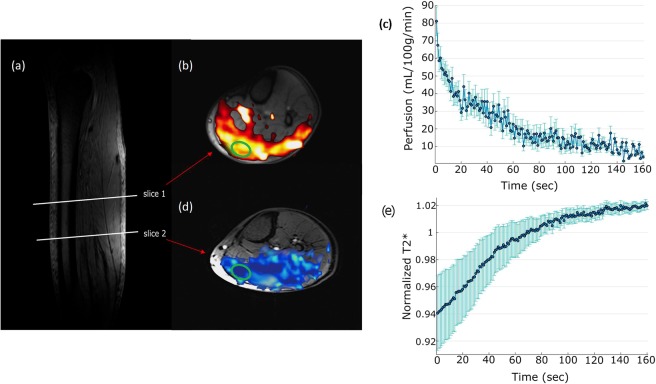
Two slice locations for perfusion and T_2_* on calf muscle (**a**). Slice 1 is perfusion slice and slice 2 is T_2_* slice. Increased perfusion activated area on corresponding perfusion slice (**b**) and average perfusion with standard deviation of all the subjects during recovery period from selected ROI (green circle) (**c**). Decreased T_2_* activated area on corresponding T_2_* slice (d) and average relative T_2_* with standard deviation of all subjects during recovery period from selected ROI (**e**).

**Figure 5:**
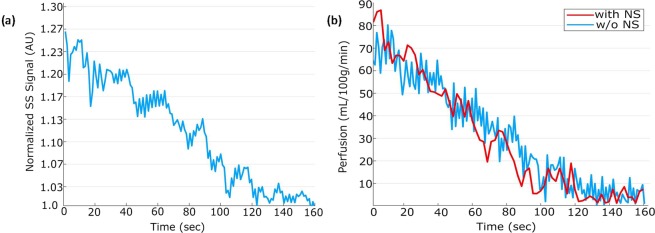
Sample slice selective (SS) signal time course from gastrocnemius muscle during recovery following exercise (**a**) and corresponding perfusion calculated without the non-selective (NS) acquisition plotted together with a sample perfusion calculated with NS acquisition (**b**).

**Figure 6:**
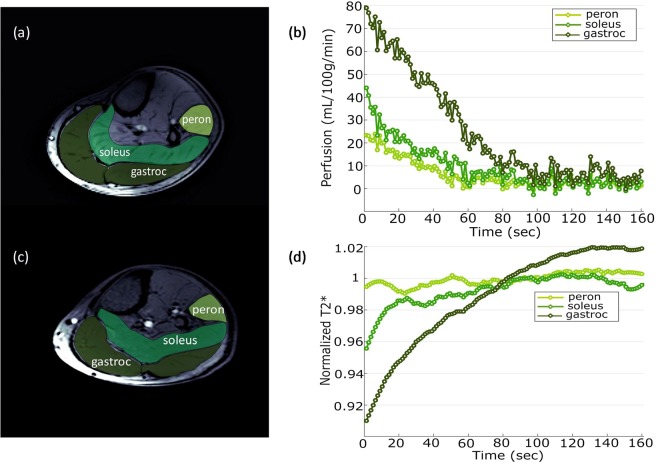
Different muscle groups in perfusion slice (**a**) and corresponding muscle perfusion (**b**) during recovery period. Same muscle groups in T_2_* slice (**c**) and corresponding muscle T_2_* (**d**) during recovery period.

Representative locations of perfusion (slice 1) and T_2_* (slice 2) are shown in Fig. 4a. At rest, the gastrocnemius muscle has the highest perfusion = 5 ± 2 mL/min/100 g. Sample SS signal time course from gastrocnemius muscle during recovery following plantar flexion exercise is shown in Fig. 5a. As SS, NS, T_2_* signals were acquired at rest and only SS, T_2_* were measured during recovery, Eq. (3) was used to estimate the NS signal during recovery period. Finally, Eq. (4) was used to calculate the perfusion. Figure 5b shows the sample perfusion time courses calculated with and without NS acquisition, which manifests that perfusion can be estimated with great accuracy even without the NS acquisition. Figure 4b,c show the perfusion results from plantar flexion exercise. Quantitative perfusion maps are overlaid on the anatomical images indicating the regions of highest change from rest (Fig. 4b). Immediately after exercise, the gastrocnemius muscle showed the highest increase in perfusion. A region of interest (ROI) corresponding to the activated muscle regions identified on the perfusion maps was manually traced on the images and the mean change in perfusion is shown in Fig. 4c. Immediately after exercise, the perfusion in the most activated area of the muscle increased to 79.3 ± 9 mL/min/100 g and decreased slowly to baseline after a relatively fast initial decline during post-exercise rest. Average time constant for 50% perfusion recovery was 54.1 ± 10 s. Figure 6a,b show the synopsis of change in perfusion in different muscle groups – gastrocnemius, soleus and peroneus muscles within calf muscle during recovery from exercise. The perfusion in soleus and peroneus muscles are not affected as much as gastrocnemius muscle due to plantar flexion exercise protocol used in this study.

Sample T_2_* curve fitting from one subject at rest is shown in Fig. S.3 (Supplementary Information). Figure 4d shows overlay of change in T_2_* after plantar flexion exercise indicating regions of muscle activation. Change in T_2_* was determined from the difference between the first and last T_2_* map generated during the recovery period. A region of interest (ROI) was selected to observe the relative change of T_2_* (normalized by the resting T_2_*) and is shown in Fig. 4e. T_2_* decreased by 6 ± 3% immediately after exercise and recovered as the subjects rested. At the end of data acquisition time, T_2_* did not return to baseline (resting value) and was still off by 2 ± 0.7%. Average time constant for 50% recovery from minimum value to this peak value was 68.5 ± 7 s. Figure 6c,d show the synopsis of change in T_2_* in different muscle groups- gastrocnemius, soleus and peroneus muscles within calf muscle during recovery from exercise. It shows that T_2_* of soleus and peroneus muscles are not affected as much as gastrocnemius muscle and return approximately to baseline while gastrocnemius muscle T_2_* remains elevated by the end of acquisition time. Table 1 shows perfusion and T_2_* values from the most activated muscle area due to exercise i.e. gastrocnemius muscle of all subjects.

**Table 1:** Perfusion and T_2_* values from gastrocnemius muscle of all the subjects.

Subject	Perfusion		T_2_*		
	Peak flow (mL/min/100 g)	50% Recovery time constant (s)	Baseline T_2_* (ms)	Minimum relative T_2_*	50% Recovery time constant (s)
1	83.1	44.5	20.49	0.96	67.2
2	88.6	66.1	21.23	0.99	58.6
3	70.3	41.9	23.27	0.94	64.3
4	84.2	49.3	24.58	0.91	71.1
5	72.6	45.6	19.73	0.96	73.7
6	91.1	71.1	22.25	0.88	81.6
7	67.8	51.2	18.31	0.97	65.4
8	77.1	56.2	17.92	0.95	69.4
9	76.3	60.7	23.4	0.90	70.8
mean ± SD	79.3 ± 9	54.1 ± 10	21.1 ± 3	0.94 ± 0.03	68.5 ± 7

## Discussion

We have demonstrated the feasibility of simultaneous quantification of changes in perfusion and T_2_* in lower leg following plantar flexion exercise. The sequence was tested and validated in phantoms before *in vivo* studies. Perfusion measurement without the NS acquisition was also compared with the one measured with the NS acquisition. The temporal resolution of the sequence allowed us to measure dynamic changes in perfusion and T_2_* as the muscle recovered from the submaximal exercise. We used plantar flexion exercises because it is a simple exercise protocol and physiologically more relevant than reactive hyperemia. The changes in post exercise perfusion and T_2_* values is in general agreement with values from previous reported studies. This pulse sequence and exercise protocol provides a unique ability to investigate different aspects of muscle metabolic function (i.e., regional perfusion and tissue blood oxygenation). Although some studies reported simultaneous measurement of perfusion and T_2_* weighted signal at 7T^22,23^, but none of them measured quantitative T_2_*. T_2_* weighted signal, which can also be termed as BOLD signal^24,25^, is dependent on the change in T_2_* as well as the initial signal intensity, which is influenced by parameters such as inflow, baseline drifts and changes in T_1_^25^. Measurement of T_2_* enables to separate the oxygenation related changes^25–27^. Moreover, the advantage of our current technique compared to the other available techniques is higher temporal resolution, which is vital for measuring notable changes in these metabolism-related parameters following exercise (Fig. 5b), and the fact that radial readout provides more flexibility in quantification of perfusion and T_2_* as sudden twitch of the subject during measurement of these parameters may result in faulty measurement, which can be overcome by radial readout and the subject feels more comfortable (Fig. S.4 in Supplementary Information).

Exercise increased the blood flow to the stimulated tissue. Perfusion measurement without the NS acquisition was compared with the one measured with NS acquisition from one subject *in vivo* during recovery following exercise, which shows that our current technique can measure perfusion with excellent accuracy. The muscle perfusion immediately at the end of exercise increased to 79.3 ± 9 mL/min/100 g from a resting value of 5 ± 2 mL/min/100 g. Prior studies with similar exercise protocols reported a peak perfusion in the range of 60 to 84 mL/min/100 g in gastrocnemius muscle^7,28,29^, in close agreement with our results. The difference in peak perfusion could be due to different work rates during the exercise protocol and selection of ROI. Lower perfusion values have been reported following reactive hyperemia^30^. This is expected because contraction elicits exercise dependent metabolic demand and increased flow directed to capillaries supplying the metabolically active tissue, whereas during reactive hyperemia the flow is elevated due to reduced vascular resistance caused by ischemia. Perfusion recovery is also in agreement with previous reports with similar exercise protocol^28^. Another study reported slower recovery of perfusion after exercise^29^, similar to recovery reported following anaerobic exercise^17^. It is difficult to speculate the difference in recovery rates between studies since exercise protocol, metabolic state of the tissue under examination or biological differences between study participants may affect the recovery rate.

T_2_* at 7T is reported to be approximately 22 ms and our resting values are in agreement with previous studies^22^. However magnetic field inhomogeneity and shim quality will affect the baseline T_2_*, as another previous study reported shorter T_2_* of ~17 ms at 7T^31^. We normalized the post exercise recovery T_2_* in each subject with the baseline T_2_* values to eliminate these scanner dependent variations and allow for comparison between subjects. A lower value of T_2_* is observed immediately after exercise and indicates deoxygenation of activated muscle; i.e., a decrease in venous oxygen saturation. As T_2_* starts to recover it goes to a peak value of approximately 2% higher than the baseline (resting value). Previous studies reported similar kind of T_2_* response during recovery following exercise^29^. This is also in agreement with BOLD studies in which the BOLD signal immediately decreased at the onset of exercise, stayed low during exercise and only recovered when the muscle was allowed to rest at the completion of exercise^24^. A drop in T_2_* weighted signal during exercise followed by a rise of the T_2_* weighted signal before the end of the exercise in gastrocnemius muscle was reported in a previous study, which was probably caused by a falling pH^22^. This can also be attributed to the fact that two competing factors, increase in flow and oxygen extraction to meet increased metabolic demand due to exercise^32–34^, and rapid decrease in partial oxygen pressure in active muscle^35^ contribute to the T_2_* weighted signal change^36^. Also, activity level can impact T_2_* weighted signal^37^ and T_2_* weighted signal represents all potential contributions to signal loss, including T_1_ effects, T_2_ effects and magnetic field inhomogeneities. Following exercise, the perfusion in activated muscle is increased which also affects local blood volume, which is expected to have pronounced effect on BOLD signal. In future with carefully designed exercise paradigms it may be possible to separate the effect of T_2_* changes due to perfusion and oxygen extraction.

There are a few limitations to the existing study. In order to calculate perfusion, the SATIR approach assumes that the muscle and blood T_1_ are same^17^. Measurement of blood and muscle T_1_ at rest and during recovery post-exercise is not practical. Hence all previous reports using SATIR approach made the assumption that blood and tissue T_1_ are equal^15,17^. Different T_1_ values for muscle (T_1_ = 1.55 sec)^21^ and blood (T_1_ = 2.08 sec)^38^ have been reported at 7T. Simulation using the reported T_1_ values for muscle and blood shows that the error introduced in perfusion measurement is not expected to exceed 7%. Another issue of assuming constant T_1_ throughout the recovery period post-exercise, as implemented by SATIR approach, may affect perfusion estimation as T_1_ is expected to change due to the change in blood oxygenation levels. The maximum TE for multi echo GRE acquisition should be ≥ T_2_*. Our maximum TE affected T_2_* quantification with a maximum error <4%, which was validated in phantom experiment. Also, some recent works showed that muscle metabolic and oxidative capacity varies along the length of the muscle even within the same muscle group^39^, which was also observed as a gradient in T_2_* weighted signals between two distal slice locations following exercise^23^. This suggests that even though the T_2_* slice should have similar kind of T_2_* response as the perfusion slice, but there might still be a gradient between the actual numbers. We did not quantify the actual work done during the exercise protocol. Metabolic demand for the tissue provides a feedback signal for increase in blood supply hence it is important to get a measure of work done by the muscles. We also used a surface coil for this study. Since there is no body coil in for 7T, we used the surface coil for both transmit/receive, which compromises the signal to noise from muscle groups at a distance from the coil. A knee coil or other volume coil placed around the calf would improve signal to noise and provide better homogeneous coverage. The plantar flexion device was not compatible with the knee coil due to size restrictions. In future, a new design of the plantar flexion device would allow the study to be conducted with the knee coil. We did not validate the perfusion measurements with an identical ASL sequence without multi-echo GRE loop. This would have required two separate exercise protocols. Difference in the exercise work rate during the two exercise protocols or fatigue can potentially make the comparison difficult. We did test in the phantom that the presence of the multi-echo loop did not affect the perfusion slice data (Fig. 3b). In the present study we evaluated the effect of perfusion loop on the T_2_* measurements and did not find any evidence that perfusion slice affects the measurement of T_2_* for at slice 3 cm distal location.

## Conclusion

This study demonstrates the ability to simultaneously quantify skeletal muscle perfusion and T_2_*, both at rest and dynamically during post exercise recovery in calf muscle at 7T using radial acquisition at high temporal resolution. An interleaved golden angle radial acquisition pulse sequence was implemented for the study which helps reduce bulk motion artifacts. The temporal and spatial resolution of the protocol was sufficient to measure changes in metabolism-related parameters post exercise. Regulated plantar flexion exercise isolates the calf muscles and might provide valuable insight into pathophysiological processes independent of impaired heart function. Accordingly, the information provided by this technique may prove to be very valuable in understanding muscle metabolism in healthy subjects as well as patient populations who experience mobility disability such as MS, PD, or older adults. This later observation will be an important next step in this line of research.

## Supplementary information

Supplementary material.
