# Supplementary material for: Simultaneous Measurement of Perfusion and T2* in Calf Muscle at 7T with Submaximal Exercise using Radial Acquisition
Source: Sci Rep. 2020 Apr 14;10:6342. doi: 10.1038/s41598-020-63009-4 (PMC7156440; doi:10.1038/s41598-020-63009-4)
Supplement: Supplementary file 1 — Supplementary material. [file 41598_2020_63009_MOESM1_ESM.pdf]

Simultaneous Measurement of Perfusion and T_2* in Calf Muscle at 7T with Submaximal Exercise using Radial Acquisition.

Sultan Z. Mahmud (szm0131@auburn.edu) [1,*]
L. Bruce Gladden (gladdlb@auburn.edu) [2]
Andreas N. Kavazis (ank0012@auburn.edu) [2]
Robert W. Motl (robmotl@uab.edu) [3]
Thomas S. Denney (dennets@auburn.edu) [1]
Adil Bashir (azb0117@auburn.edu) [1]

[1] Department of Electrical and Computer Engineering, Auburn University, Auburn, AL 36849, USA.

[2] School of Kinesiology, Auburn University, Auburn, AL 36849, USA.

[3] Department of Physical Therapy, University of Alabama at Birmingham, Birmingham, AL 35294, USA.

Supplementary Information

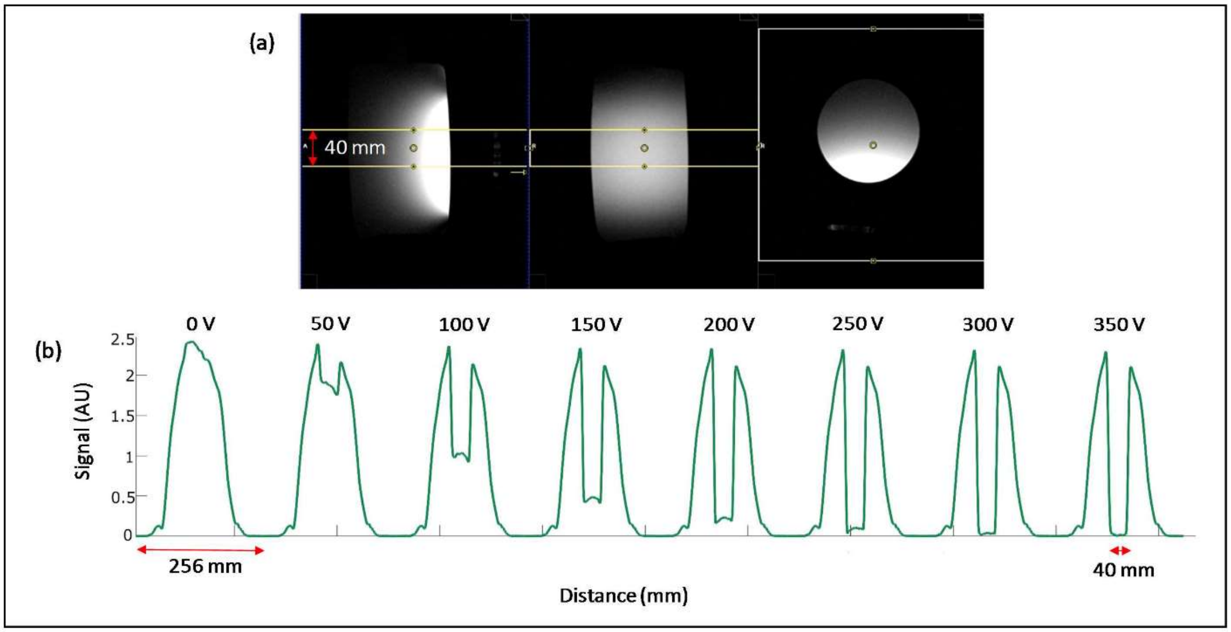

Figure S.1: (a) Representative coronal, sagittal and axial images of the phantom (cylindrical phantom with diameter =110 mm and length= 300 mm). The slice location is also shown on the images. 1D ISIS pulse sequence with the readout direction from top to bottom i.e. along the length of the cylindrical phantom was used to calibrate the adiabatic inversion pulse. Inversion pulse was turned ON/OFF for every alternate acquisition. If a complete 180° inversion is achieved the signal from the selected slice should be eliminated when even and odd acquisitions are summed. Other imaging parameters were FOV=256 mm, slice thickness = 40 mm, TR = 2 sec, TE = 6 ms, averages = 8. (b) 1D images of the object. As the voltage of the inversion pulse was incremented the signal from the selected slice decreases. The signal is completely suppressed for voltages greater then 250V for the adiabatic inversion pulse. The 1D profile represents the summation of spins over the transverse depth of the phantom demonstrating that the complete inversion is achieved over the phantom.

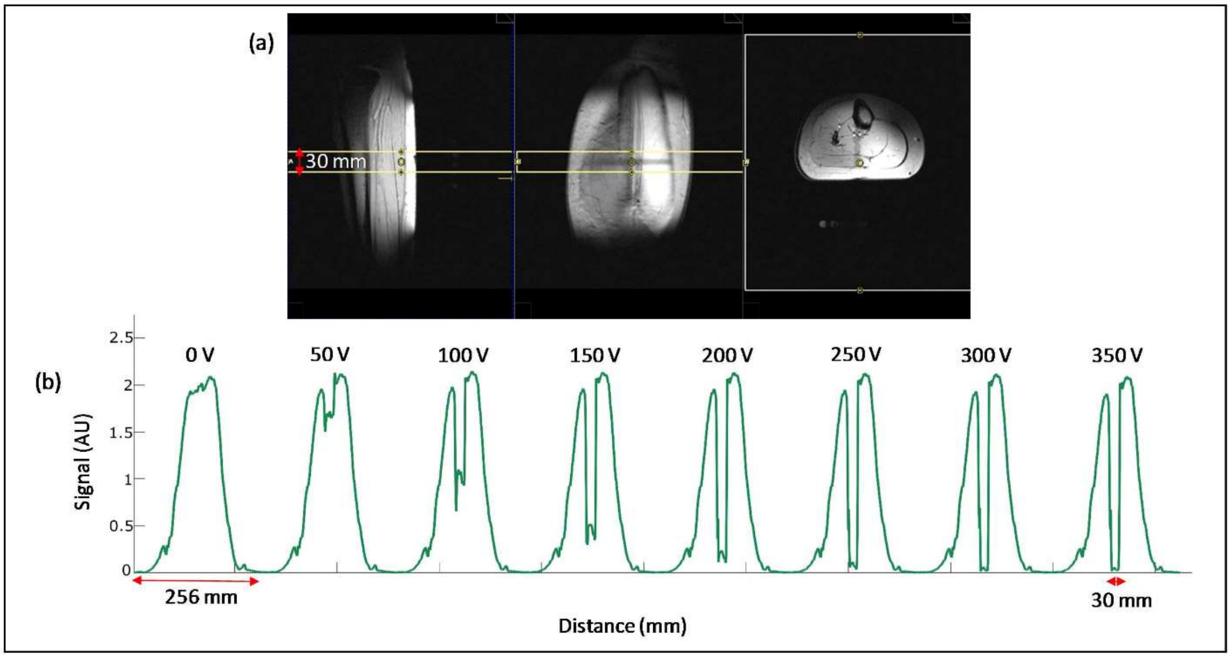

Figure S.2: (a) Representative coronal, sagittal and axial images of the calf. 1D ISIS sequence and imaging protocol as described in Fig S.1, with slice thickness=30 mm, was used to determine the calibrate the adiabatic inversion pulse. (b) 1D profiles as a function of pulse voltage. Complete inversion over the transverse extent of the leg is achieved for RF pulse voltages over 250V.

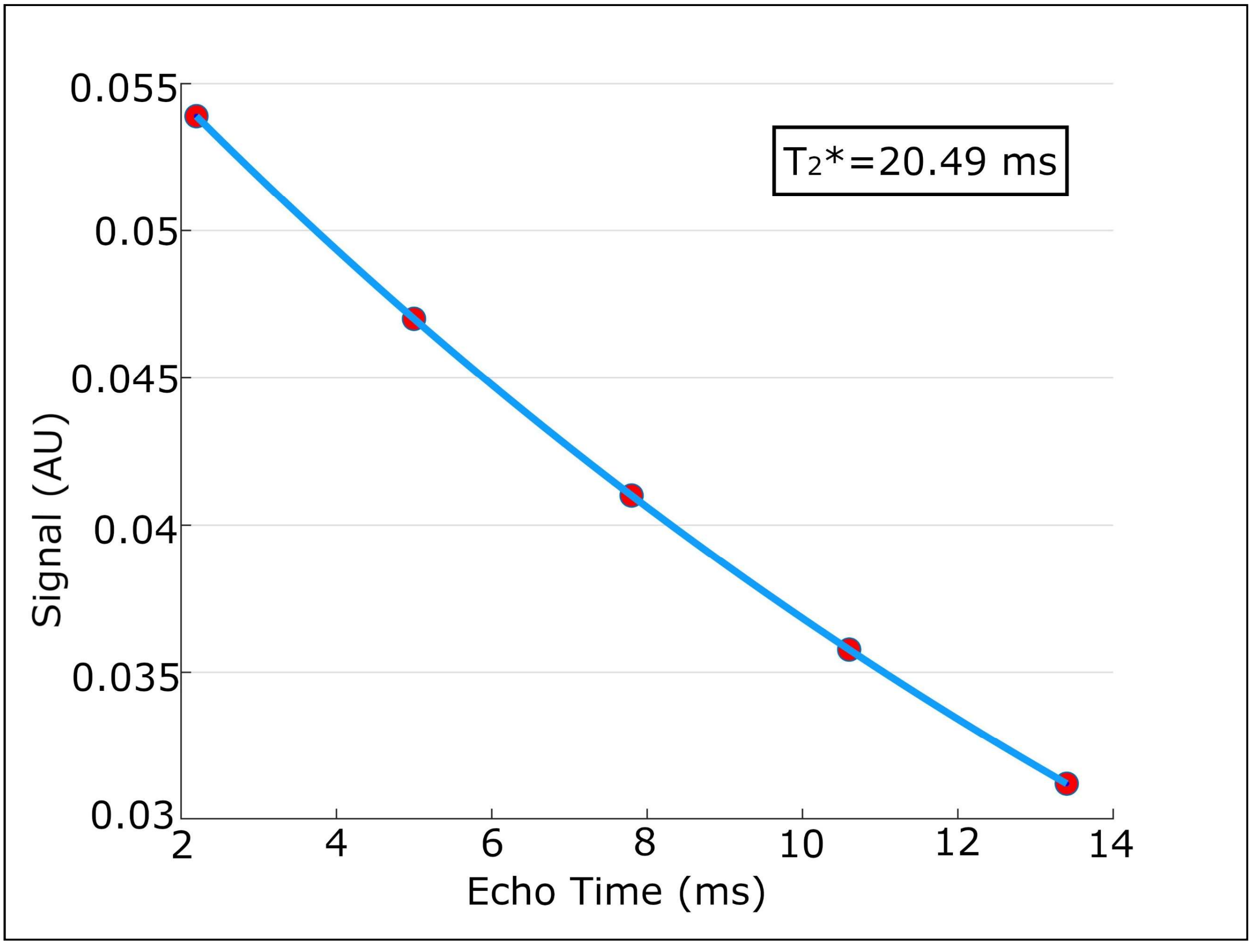

Figure S.3: Representative T2* curve fitting from one subject at rest.

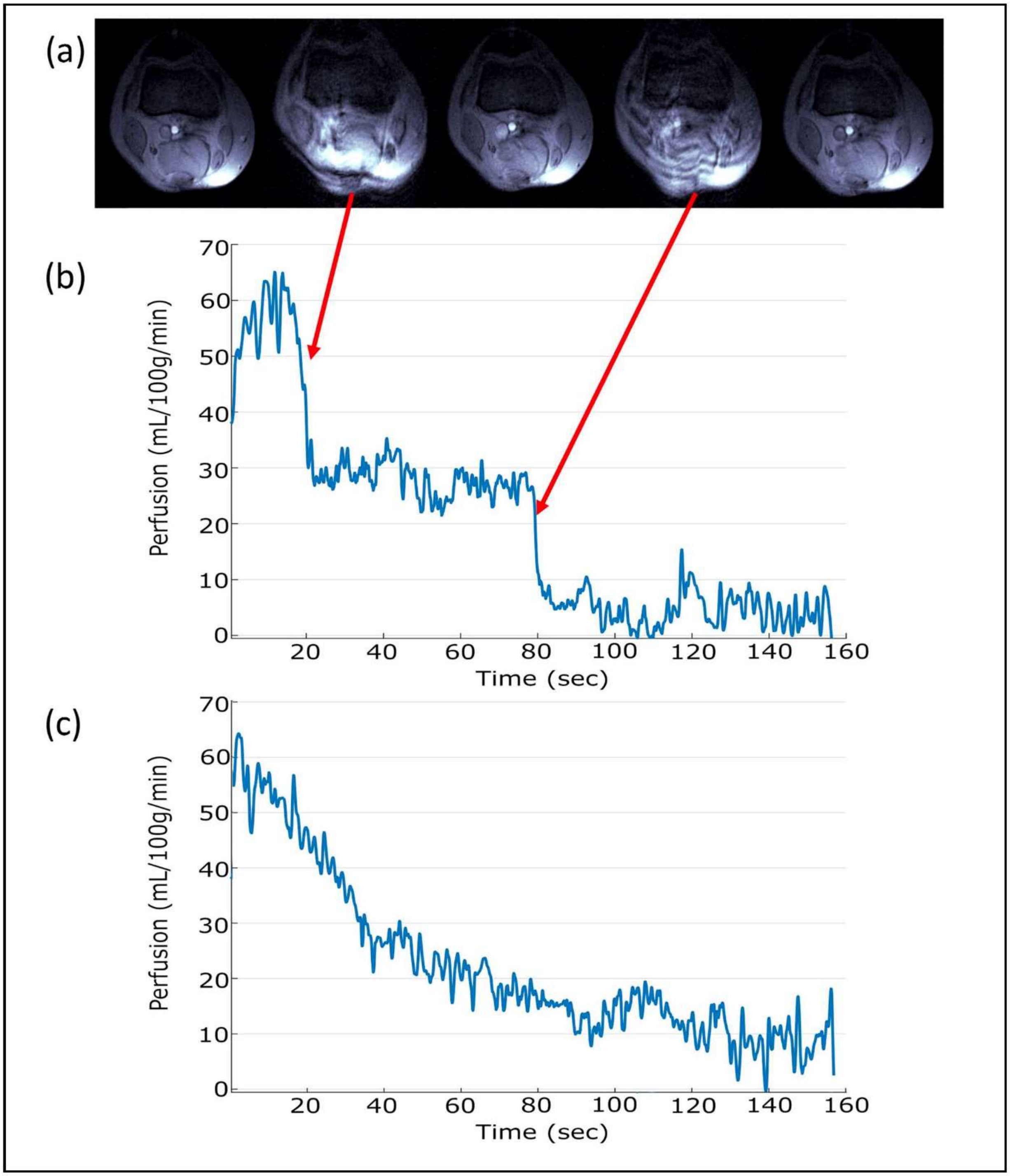

Figure S.4: Sample leg image time series during recovery following exercise is shown in (a) and the perfusion calculated using Cartesian readout during recovery period is shown in (b). We can see that when the subject twitched the leg (2nd and 4th images from the left in (a)), perfusion measurement resulted in faulty estimation, which was overcome by radial acquisition (c).
